# Supplementary material for: Switchable Exchange Bias Resulting From Correlated Domain Structures in Orthogonally Coupled Antiferromagnet/Ferromagnet van der Waals Heterostructures
Source: Small. 2025 Sep 12;21(42):e06284. doi: 10.1002/smll.202506284 (PMC12547998; doi:10.1002/smll.202506284)
Supplement: Supplementary file 1 — Supporting Information [file SMLL-21-e06284-s001.pdf]

NANO · MICRO
small

Supporting Information

for *Small*, DOI 10.1002/smll.202506284

Switchable Exchange Bias Resulting From Correlated Domain Structures in Orthogonally Coupled Antiferromagnet/Ferromagnet van der Waals Heterostructures

Aditya Kumar, Sadeed Hameed, Thibaud Denneulin, Aravind Puthirath Balan, Joseph Vas, Kilian Leutner, Lei Gao, Olena Gomonay, Jairo Sinova, Rafal E. Dunin-Borkowski and Mathias Kläui**

Supporting Information

Switchable Exchange Bias Resulting from Correlated Domain Structures in Orthogonally Coupled Antiferromagnet/Ferromagnet van der Waals Heterostructures

Aditya Kumar, Sadeed Hameed, Thibaud Denneulin, Aravind Puthirath Balan, Joseph Vas, Kilian Leutner, Lei Gao, Olena Gomonay, Jairo Sinova, Rafal E. Dunin-Borkowski, Mathias Kläui**

Contents

SI 1 Atomic Force Microscopy of CrSBr/Fe_3GeTe_2 Heterostructures 2

SI 2 Raman spectroscopy 2

SI 3 Additional anomalous Hall effect measurements in CrSBr/FGT vdW heterostructure 3

SI 4 Off-axis electron holography of the CrSBr/FGT cross-section 6

SI 5 Modeling of the magnetic textures in the antiferromagnet-ferromagnet bilayer 8

SI 1 Atomic Force Microscopy of CrSBr/Fe_3GeTe_2 Heterostructures

To check the thickness of the exfoliated flakes used in our h-BN/CrSBr/Fe_3GeTe_2 van der Waals heterostructures, we performed atomic force microscopy measurements on two representative samples. The line scans and corresponding height profiles are presented in Figure SI1. These measurements indicate that the Fe_3GeTe_2 (FGT) flakes in D1 and D2 are approximately 87 nm and 97 nm thick, respectively. The CrSBr flakes in the same samples were measured to be 30 nm and 63 nm thick, respectively. The atomic force microscopy images and corresponding line profiles are shown in Figure SI1.

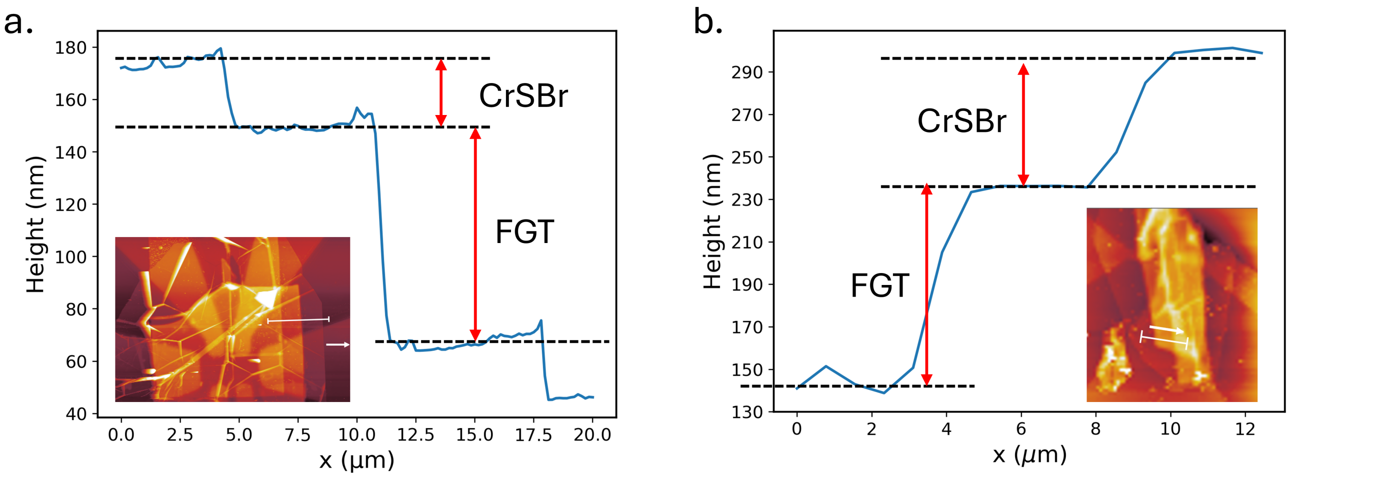

Figure SI1: Atomic force microscopy measurements of h-BN/CrSBr/FGT van der Waals heterostructures for (a) D1 and (b) D2. Insets show the corresponding atomic force microscopy scan images. In D1, the thicknesses of the FGT and CrSBr layers are 87 ± 2 nm and 30 ± 2 nm, respectively. In D2, the FGT layer is approximately 97 ± 2 nm thick, while the CrSBr layer is 63 ± 2 nm.

SI 2 Raman spectroscopy

Raman spectroscopy was performed on both the FGT and CrSBr flakes to assess their structural quality. The corresponding Raman spectra for both materials are shown in Figure SI2.

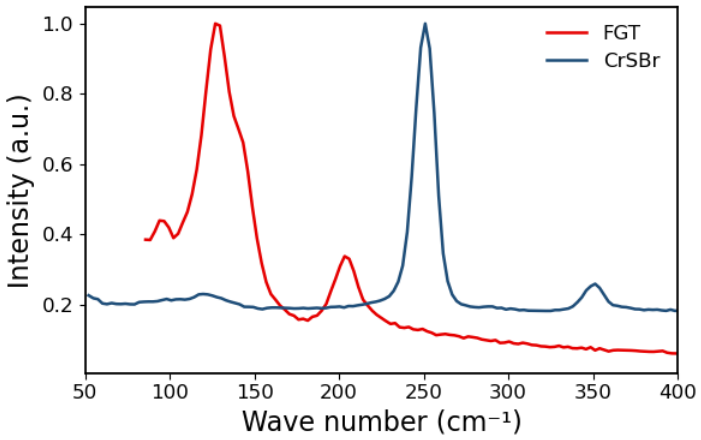

Figure SI2: Raman spectra of bulk crystal of Fe_3GeTe_2 and exfoliated flake of CrSBr.

SI 3 Additional anomalous Hall effect measurements in CrSBr/FGT vdW heterostructure

We confirm the reproducibility of the asymmetric hysteresis loop and exchange bias in the CrSBr/FGT van der Waals heterostructure. The zero-field-cooled (ZFC) hysteresis loop measured for D2 exhibits similar asymmetry under both +2.5 T and −2.5 T preset fields. Figure SI3 shows the anomalous Hall effect (AHE) hysteresis loops measured after positive, negative, and zero preset fields. Compared to D1, the exchange bias and coercivity observed in D2 are slightly reduced, likely due to its larger thickness. The ZFC loop displays gradual switching on both sides, resulting in no measurable exchange bias.

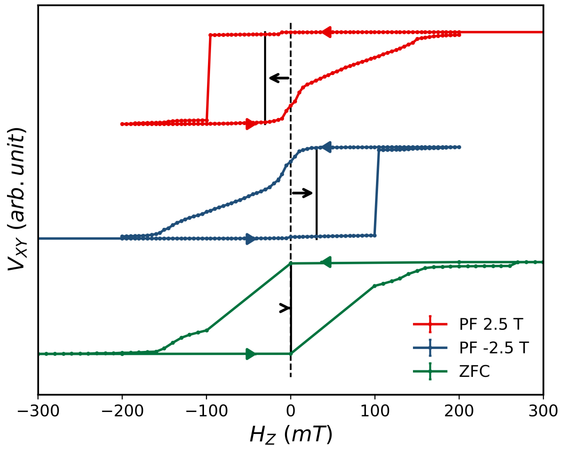

Figure SI3: AHE hysteresis loop measured for sample 2 at ZFC, 2.5 T preset-field and -2.5 T preset field.

Negative preset-field measurements for D1 also exhibit similar hysteresis loop shapes, as shown in Figure SI4a and b. The temperature dependence of coercivity in the CrSBr/FGT van der Waals heterostructure follows a similar trend for both positive and negative preset fields, as shown in Figure SI4c. Figure SI4d further demonstrates the persistence of hysteresis loop asymmetry under negative preset fields. Notably, the positive coercive field (H_C^+) follows a trend comparable to that of the exchange bias field (H_{EB}) as a function of temperature, while the negative coercive field (H_C^-) displays a conventional ferromagnetic temperature dependence.

The dependence of exchange bias on the magnitude of the preset field in the CrSBr/FGT heterostructure is presented in Figure SI4e. These measurements confirm the reproducible and switchable nature of exchange bias in this system. The exchange bias field increases with preset field strength and saturates around 1 T.

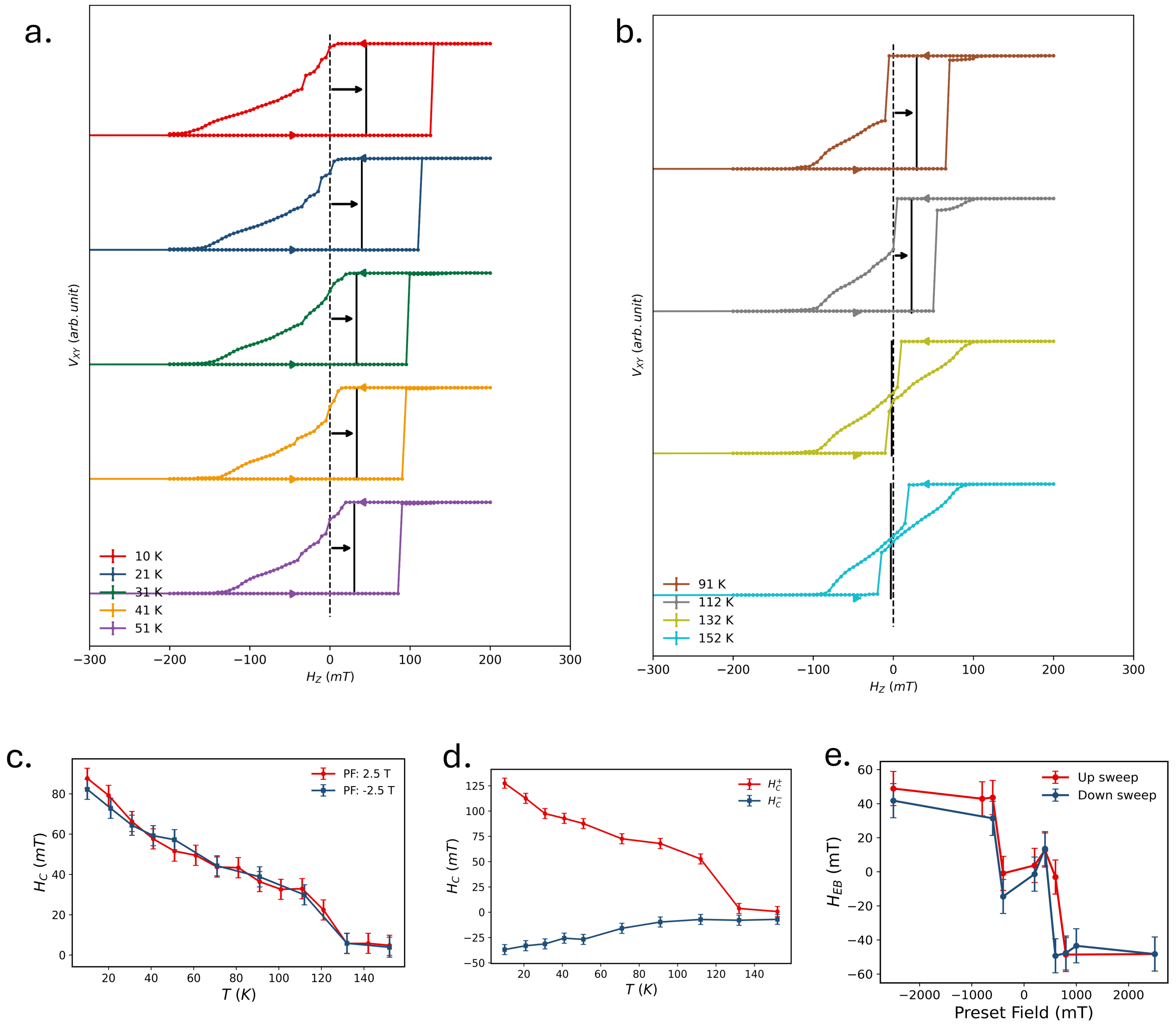

Figure SI4: (a–b) Out-of-plane hysteresis loops measured at temperatures ranging from 10 K to 152 K after applying a negative preset field of −2.5 T for D1. The loops are vertically offset for clarity. (c) Temperature dependence of coercivity for D1. (d) Positive and negative coercive fields plotted as a function of temperature under a −2.5 T preset field. (e) Exchange bias as a function of preset field strength, highlighting its switchability.

The temperature dependence of the coercive field and exchange bias exhibit closely correlated trends, as shown in Figure SI5a. Notably, the temperature profile of the abrupt switching field aligns nearly identically with that of the exchange bias. This correlation arises because the gradual switching field displays expected monotonic decrease with temperature, while abrupt switching field predominantly contributes to the exchange bias.

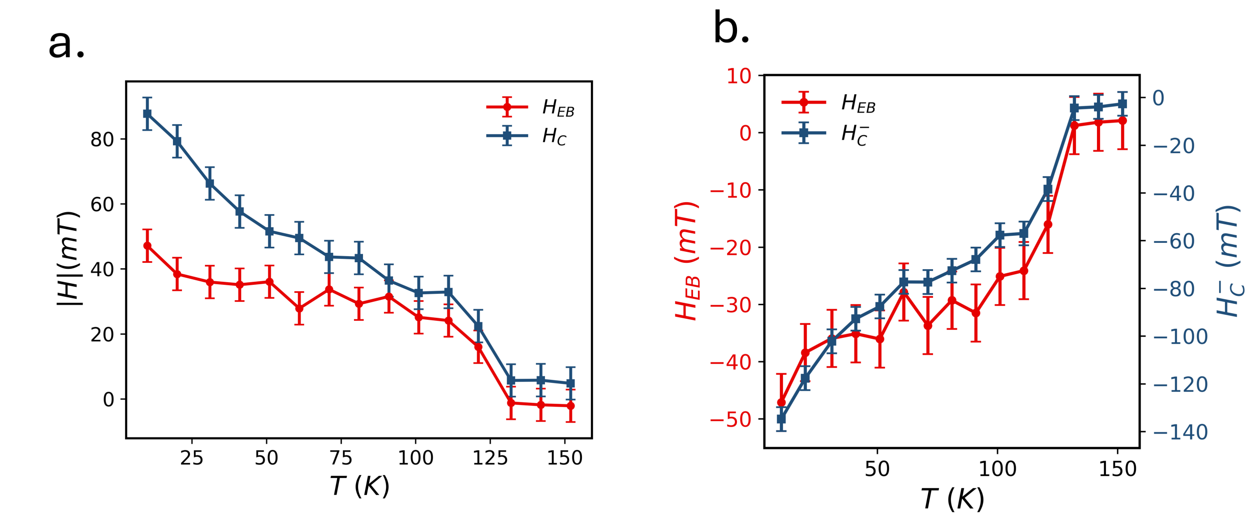

Figure SI5: **Temperature-dependence of exchange bias and coercive field.** (a) Exchange bias (H_{EB}) and coercive field (H_{C}) as a function of temperature, illustrating the gradual reduction with increasing temperature. (b) Exchange bias and coercive field on the negative field side, where monodomain switching occurs, as a function of temperature.

The nature of the exchange bias is further investigated by measuring minor loops with and without the application of a preset-field. Figure SI6a shows minor loops recorded after applying a 2.5 T preset-field. Loops 1 and 2 display the expected behavior, exhibiting asymmetric switching and a clear exchange bias. However, loops 3 and 4 show no discernible deviation from the saturated state, indicating a frozen magnetic configuration. In contrast, loops 2–8 in Figure SI6b exhibit conventional ferromagnetic behavior without exchange bias. These observations suggest that the out-of-plane (OOP) saturated state of CrSBr following a preset-field remains metastable even after the preset-field has been removed. This OOP saturated state, induced by the preset-field, persists until the magnetization reversal of FGT, when it disrupts the interfacial coupling, thereby breaking the metastable condition. Once CrSBr goes to in-plane canted antiferromagnetic state we see conventional minor loops for a ferromagnet.

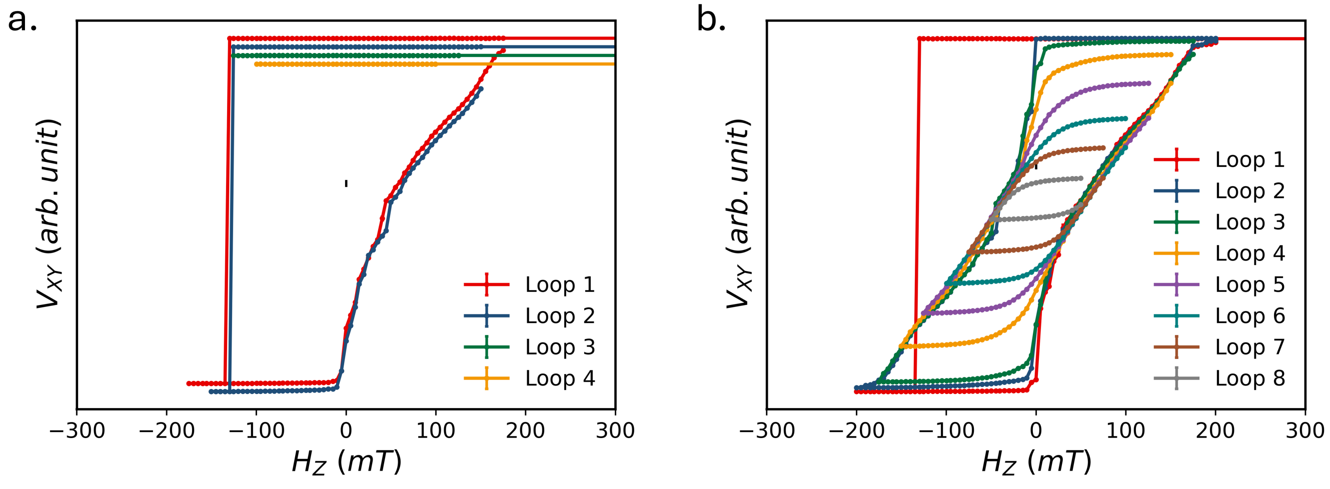

Figure SI6: Minor AHE loops measured in the CrSBr/FGT van der Waals heterostructure. (a) Minor loops recorded after applying a preset-field of 2.5 T. The measured loops are vertically offset for better legibility. (b) The first loop is measured immediately after applying the preset field, followed by consecutive minor loops measured sequentially without reapplying the preset-field.

SI 4 Off-axis electron holography of the CrSBr/FGT cross-section

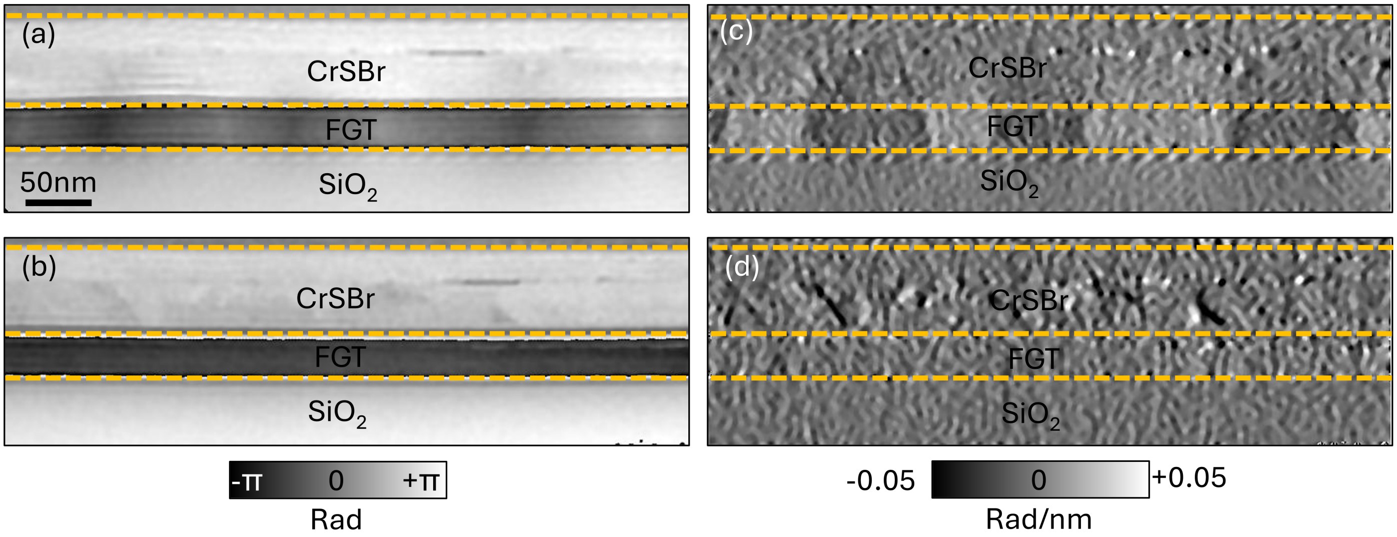

Figure SI7: (a, b) Raw phase images obtained via off-axis electron holography. (a) Domain structure after zero-field cooling. (b) Saturated state following the application of a 1 T magnetic field along c axis. (c, d) Corresponding horizontal phase gradient images highlighting the local magnetic induction direction.

Off-axis electron holography is a powerful transmission electron microscopy (TEM) technique capable of resolving magnetic structures with spatial resolution on the order of 10 nm.[1, 2] This method captures the phase shift of an electron wave that has passed through the sample. The electron wave interferes with a reference wave traveling in vacuum to form a hologram, from which the phase shift is reconstructed via Fourier transform. The local magnetic field distribution is then obtained by computing the gradient of the phase image.

For this measurement, a cross-section lamella of the CrSBr/FGT van der Waals heterostructure was fabricated using focused ion beam (FIB) milling along the *bc*-plane of CrSBr. The lamella was cooled to 95 K to induce magnetic ordering and was measured at zero magnetic field. Figure SI7a shows the raw phase map obtained at 95 K and 0 mT, while Figure SI7c shows the corresponding horizontal phase gradient. These images reveal the presence of magnetic domains in the zero field cooled (ZFC) state. After applying a 1 T OOP magnetic field, the domain pattern vanishes, as evidenced in the phase and gradient maps shown in Figures SI7b and d.

To further resolve the internal structure of the magnetic domains, we processed the phase map obtained prior to field application. A background phase image acquired at 300 K (above the Curie temperature) was subtracted from the 95 K phase map to eliminate non-magnetic contributions. The resultant magnetic phase image is shown in Figure SI8a. The structure of the flux closure domain is now more clearly visible. After applying a Gaussian noise filter (Figure SI8b), the phase gradient was calculated and color-coded to visualize the magnetic induction field direction, as shown in Figure SI8c.

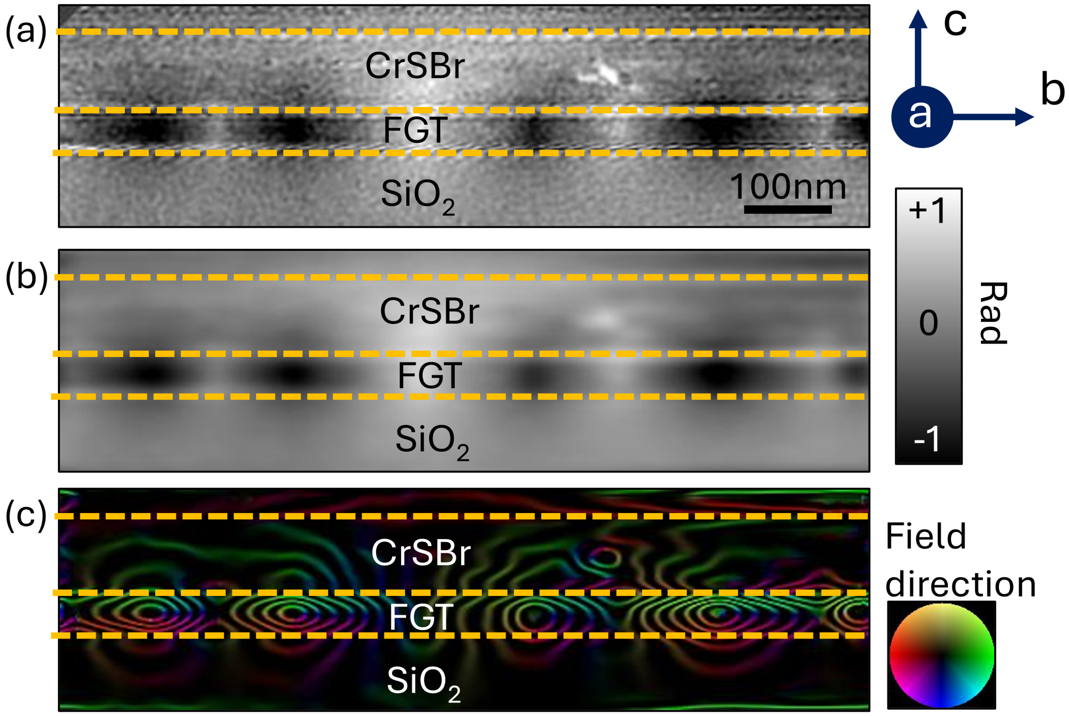

Figure SI8: (a) Magnetic phase image of the zero-field cooled state obtained after subtraction of non-magnetic variations using a phase image acquired above the Curie temperature. (b) Same image after application of a Gaussian filter. (c) Color-coded map that shows the direction of the magnetic induction field B with a contour spacing of $2\pi/30$.

To study the evolution of magnetic domains toward saturation, we performed history-dependent off-axis electron holography on the *bc* cross-section of the CrSBr/FGT van der Waals heterostructure. Phase maps, obtained after subtracting the room-temperature reference image, are shown in Figure SI9. The corresponding magnetic induction maps for selected regions (highlighted by white rectangles) are presented in Figure 4 of the main text. Image artifacts in the right portion of the field of view hindered reliable calculation of magnetic induction in that region.

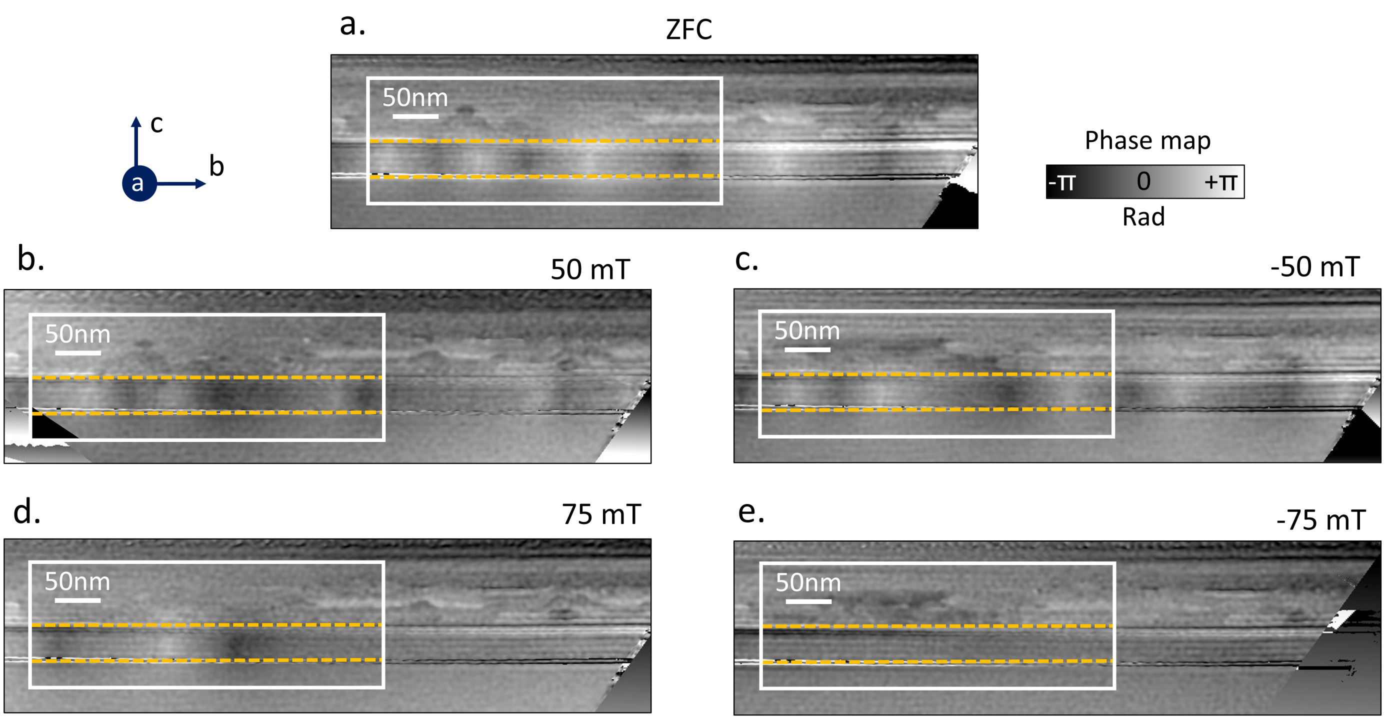

Figure SI9: **Phase maps of history-dependent field imaging.** Phase maps obtained by subtracting the room temperature phase map from those acquired at 95 K. The phase maps were recorded at zero applied field after sequential out-of-plane (OOP) magnetic field application. White boxes indicate regions further processed to estimate the magnetic induction map shown in Figure 4 of the main text. Measurement artifacts present on the right side of the images hindered reliable extraction of the field induction map. Images (a)–(e) were recorded in chronological order: (a) zero-field-cooled state; (b) after +50 mT OOP field; (c) after −50 mT OOP field; (d) after +75 mT OOP field; (e) after −75 mT OOP field. Scale bar: 50 nm.

SI 5 Modeling of the magnetic textures in the antiferromagnet-ferromagnet bilayer

To interpret our observations, we develop a model based on the minimization of the free energy of the antiferromagnet (AFM)-ferromagnet (FM) bilayer with the partial thicknesses of the FM and AFM layers t_{F} and t_{AFM}, correspondingly. For computational purposes, we also introduced a thin interfacial layer with the small but finite thickness $t_{\mathrm{int}} \ll t_{\mathrm{F}}, t_{\mathrm{AFM}}$. The free energy $F = F_{\mathrm{AFM}} + F_{\mathrm{FM}} + F_{\mathrm{int}}$ includes three terms: energy density per unit area of the AFM,F_{AFM}, and the FM, F_{FM}, layers, and interaction energy, F_{int}. The energy of the FM layer depends on the ferromagnetic magnetization $\mathbf{M}_{\mathrm{F}}$:

$$F_{\mathrm{FM}} = \int_{-t_{\mathrm{F}}}^{-t_{\mathrm{int}}/2} \left[-\frac{1}{2M_{\mathrm{F}}} H_{\mathrm{F}} M_{Fz}^2 + \frac{1}{2} A_{\mathrm{F}} \left(\nabla \mathbf{M}_{\mathrm{F}}\right)^2 - \mathbf{H} \cdot \mathbf{M}_{\mathrm{F}} \right] dz, \qquad (1)$$

where H_{F} is an anisotropy field that aligns FM magnetization out of film plane (along z axis), A_{F} is a ferromagnetic stiffness, and $\mathbf{H}$ is an external magnetic field.

The energy of the AFM layer depends on the Néel vector $\mathbf{n} = \mathbf{M}_1 - \mathbf{M}_2$ and magnetization $\mathbf{m} = \mathbf{M}_1 + \mathbf{M}_2$, where $\mathbf{M}_{1,2}$ are magnetic sublattices:

$$\begin{aligned} F_{\mathrm{AFM}} \;=\; & \int_{t_{\mathrm{int}/2}}^{t_{\mathrm{AF}}} \left\{ \frac{1}{2M_{\mathrm{s}}} H_{\mathrm{ex}} \mathbf{m}^2 + \frac{1}{2M_{\mathrm{s}}} H_{\mathrm{c}} (n_z^2 + m_z^2) \right. \\ & \left. - \frac{1}{2M_{\mathrm{s}}} \left(H_{\mathrm{b}} - H_4 \frac{\mathbf{m}^2}{M_{\mathrm{s}}^2} \right) (n_x^2 + m_x^2) + \frac{1}{2} A_{\mathrm{AF}} \left[(\nabla \mathbf{n})^2 + (\nabla \mathbf{m})^2 \right] - \mathbf{H} \cdot \mathbf{m} \right\} dz, \end{aligned} \qquad (2)$$

where H_{ex} is the intersublattice exchange that keeps sublattice magnetizations $\mathbf{M}_{1,2}$ antiparallel in absence of the magnetic field, $H_{\mathrm{b}}, H_{\mathrm{c}} > 0$ are anisotropy fields that favours orientation of the Néel vector along b

axis, A_{AF} is the stiffness of AFM, $|\mathbf{M}_{1,2}| = M_s/2$. The term with H_4 describes an additional contribution to anisotropy related to the canting of the magnetic sublattices (parametrized with the magnetization $\mathbf{m}$). This term is usually neglected when modeling AFMs with strong intersublattice exchange due to the negligible value of canting. In the case of CrSBr, the value of the intersublattice exchange is small, as it couples magnetic moments lying in different layers. As a result, the exchange coupling can compete with the external magnetic field. Thus, the term with H_4 is significant.

Interaction energy is modeled as follows:

$$F_{\mathrm{int}} = \int_{-t_{\mathrm{int}}/2}^{t_{\mathrm{int}}/2} \left(-J_n \mathbf{M}_F \cdot \mathbf{n} - J_m \mathbf{M}_F \cdot \mathbf{m}\right) dz, \tag{3}$$

where the constants J_n and J_m describe the exchange coupling between AFM and FM layers. We assume that FM couples more strongly with one AFM sublattice than the other. This is related to the layered structure of van der Waals materials and the weak coupling between atomic planes.

The distribution of magnetic moments is calculated by minimizing the free energy F (analytical for the homogeneous case and numerical for the texture) with additional limitations that ensure the constant length of magnetic vectors. $|M_F| = M_F$, and $\mathbf{m}^2 + \mathbf{n}^2 = M_s^2$.

We start from the analysis of the equilibrium states of the single-layered AFM (CrBSr) system. If the magnetic field is applied parallel to easy magnetic axis b, the AFM shows step-like transition between the AFM ordered ($\mathbf{n}\|b$, $\mathbf{m}$=0) and FM phase ($\mathbf{m}\|b$, $\mathbf{n}$=0) (see Fig. 1e of the main text). The AFM phase is stable in the field range $|H| \leq 2\sqrt{H_{\mathrm{ex}}H_b}$ (i.e., below the spin-flop field), while the FM phase becomes stable at $|H| \geq H_{\mathrm{ex}}$ (above the spin-flip field). In case of small exchange coupling, $H_{\mathrm{ex}} < 2H_b$, AFM and FM phases can coexist in the range $H_{\mathrm{ex}} < |H| < 2\sqrt{H_{\mathrm{ex}}H_b}$. This can explain small hysteretic loops observed close to the spin-flip transition (Fig. 1e of the main text).

Next, we consider reorientation in the magnetic field parallel to the c axis. Without an external field, the energy potential (2) has two minima. One minimum corresponds to the AFM phase ($\mathbf{n}\|b$, $\mathbf{m}$=0), and the other corresponds to the FM phase ($\mathbf{m}\|c$, $\mathbf{n}$=0). However, the second minimum is local and is separated by a high energy barrier from the ground AFM state. The magnetic field H causes the canting of magnetic sublattice ($\mathbf{n}\|b$, $\mathbf{m}\|c$, $m \neq 0$), and it causes an abrupt transition into the FM state above a certain threshold (see Figure SI10), also consistent with the reported magnetization vs field dependence [3]. Cycling the magnetic field above the threshold value can result in hysteretic behavior, in which the FM state, once reached, is stable down to $H_{\mathrm{cr1}} = H_{\mathrm{ex}} + H_c - H_4$ as shown in Fig.SI10. The observation of hysteretic behavior can be hindered by the formation of interphase boundaries. Such boundaries can be shifted by the external field, thereby resulting in a smooth field dependence, as illustrated in Figure 1e of the main text. However, the hysteretic behavior manifests upon cycling at relatively high field values, which effectively remove interphase boundaries from the sample. It is worth noting that the metastable FM phase is stabilized because of the fourth-order anisotropy (H_4) term.

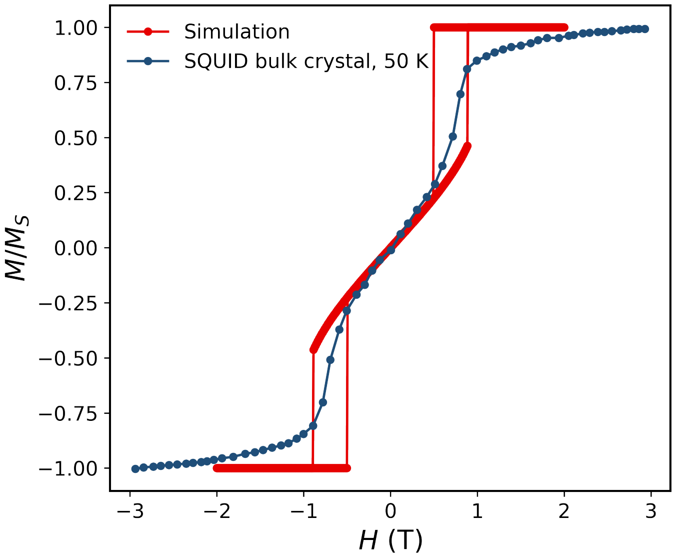

Figure SI10: Red curves show the calculated hysteresis loop of CrSBr with as assumed possible fourth-order anisotropy term. Blue curve shows magnetic moment measurement in bulk crystal by Telford et. al. at 50 K.[3]

Finally, we consider the texture of the coupled AFM/FM bilayer under the influence of a magnetic field that is aligned along the c axis. In the case of a high positive field value (state i), the ground states of both the ferromagnetic and antiferromagnetic layers are found to be homogeneous and ferromagnetic. At a high positive field value (state i) the ground states of both FM and AFM layers are homogeneous and ferromagnetic. Due to the interface coupling (term with J_m in Eq.(3)) both ferromagnetic vectors $\mathbf{m}$ and $\mathbf{M}_\mathrm{F}$ are parallel to each other. This state is stable down to the values of the external field at which the effective field in AFM layer reaches zero: $H \approx H_\mathrm{cr1} - J_m M_\mathrm{F} t_\mathrm{inf}/t_\mathrm{AF}$. Because both layers are magnetically homogeneous and there are no domain walls, bilayer then switches into a stable state where both the FM and AFM layers are ferromagnetic and the magnetization points in opposite directions (parallel to the magnetic field, state iii). However, now the AFM layer is in a canted state ($\mathbf{n}\|b$, $\mathbf{m}\|c$, $m \neq 0$) and decreasing the magnetic field value causes an increase of n.

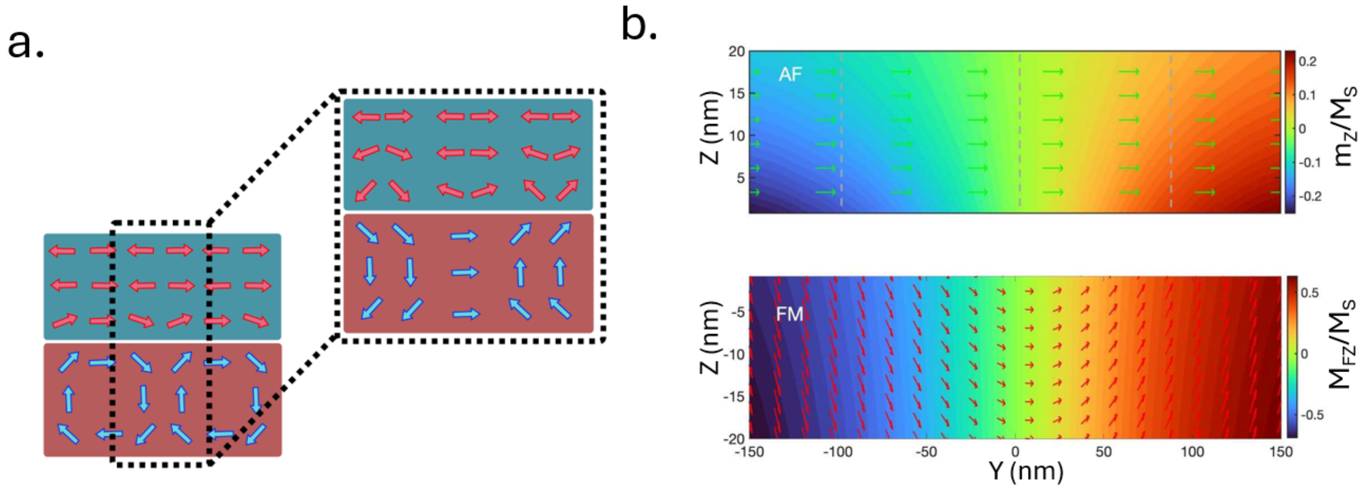

Figure SI11: (a) Schematic showing the domain wall between the two circular flux-closure domains. The schematic shows the canting of CrSBr (blue region) at different positions of the domain wall. (b) Simulated magnetization map of FGT and CrSBr, showing the canting of magnetization in CrSBr at different positions above the FGT domain wall. Green horizontal arrows in CrSBr correspond to the Néel vector.

Until now, we have only considered the effects of anisotropy and exchange. However, the OOP polarization of FM creates strong stray fields, which could be reduced by the formation of 180° domains. On the other hand, the formation of the multidomain state is separated from the homogeneous state by an energy barrier proportional to the energy of the domain walls in FM, $E^{\mathrm{F}}_{\mathrm{DW}} \propto \sqrt{A_{\mathrm{F}} H_{\mathrm{F}} M_{\mathrm{F}}} t_{\mathrm{F}}$. While this energy barrier is high enough to prevent the formation of domains in a freestanding FM layer, it can be substantially reduced in AFM/FM bilayers due to interfacial coupling. Figure SI11b shows the magnetization distribution in both layers above a domain wall, corresponding to the region indicated in Figure SI11a, which was calculated by minimizing the free energy functional F. For the numerical simulations we used the open boundary conditions and introduced the seed domain wall into a FM layer. Our simulations show that the Néel vector (green arrows) is maximal close to the center of the domain wall, where the ferromagnetic magnetization has a component parallel to the film plane. Such alignment reduced the energy of the domain wall as follows: $E^{\mathrm{F}}_{\mathrm{DW}} \to E^{\mathrm{F}}_{\mathrm{DW}} \left[1 - (J_n/H_{\mathrm{F}} M_F)(t_{\mathrm{int}}/t_{\mathrm{F}}\right]$. Consequently, coupling with the AFM layer facilitates the formation of a multidomain state (state (iv) in Fig. 4 of the main text). Further field cycling induces a domain wall shift associated with smooth magnetization variation (lines (iv) to (v) in Fig. 4 of the main text), explaining the training effect seen in Fig. 2e of the main text.

To further investigate the effect of exchange coupling between FGT and CrSBr, we model the system using micromagnetic simulations. Figure SI11b shows the simulated cross-sectional spin configuration of the CrSBr/FGT van der Waals heterostructure. The simulated region corresponding to the domain wall area is illustrated in Figure SI11a. The results reveal that the in-plane anisotropy of CrSBr, combined with interfacial exchange coupling with FGT, promotes the formation of vortex-like flux closure domains in FGT.

References

[1] P. A. Midgley, R. E. Dunin-Borkowski, *Nature Materials* **2009**, *8*, 4 271.

[2] T. Denneulin, B. Zingsem, J. Vas, W. Shi, L. Yang, M. Feuerbacher, R. E. Dunin-Borkowski, *Ultramicroscopy* **2025**, *271* 114119.

[3] E. J. Telford, A. H. Dismukes, K. Lee, M. Cheng, A. Wieteska, A. K. Bartholomew, Y.-S. Chen, X. Xu, A. N. Pasupathy, X. Zhu, C. R. Dean, X. Roy, *Advanced Materials* **2020**, *32*, 37 2003240.
